# Non-Linear Cellular Dielectrophoretic Behavior Characterization Using Dielectrophoretic Tweezers-Based Force Spectroscopy inside a Microfluidic Device

**DOI:** 10.3390/s18103543

**Published:** 2018-10-19

**Authors:** Seungyeop Choi, Kwanhwi Ko, Jongwon Lim, Sung Hoon Kim, Sung-Hun Woo, Yoon Suk Kim, Jaehong Key, Sei Young Lee, In Su Park, Sang Woo Lee

**Affiliations:** 1 Department of Biomedical Engineering, Yonsei University, Wonju 26493, Korea; sychoi0091@gmail.com (S.C.); ko.kwanhwi@gmail.com (K.K.); jongwonlim44@naver.com (J.L.); jkey@yonsei.ac.kr (J.K.); syl235@yonsei.ac.kr (S.Y.L.); 2 Department of Biomedical Laboratory Science, Yonsei University, Wonju 26493, Korea; k140017@naver.com (S.H.K.); sunghun2120@gmail.com (S.-H.W.); yoonsukkim@yonsei.ac.kr (Y.S.K.); 3 Micro and Nanotechnology Laboratory, University of Illinois at Urbana–Champaign, Urbana, IL 61801, USA

**Keywords:** dielectrophoresis, force spectroscopy, non-linear cellular DEP Behaviors, cross-over frequency, microfluidic device

## Abstract

Characterization of cellular dielectrophoretic (DEP) behaviors, when cells are exposed to an alternating current (AC) electric field of varying frequency, is fundamentally important to many applications using dielectrophoresis. However, to date, that characterization has been performed with monotonically increasing or decreasing frequency, not with successive increases and decreases, even though cells might behave differently with those frequency modulations due to the nonlinear cellular electrodynamic responses reported in previous works. In this report, we present a method to trace the behaviors of numerous cells simultaneously at the single-cell level in a simple, robust manner using dielectrophoretic tweezers-based force spectroscopy. Using this method, the behaviors of more than 150 cells were traced in a single environment at the same time, while a modulated DEP force acted upon them, resulting in characterization of nonlinear DEP cellular behaviors and generation of different cross-over frequencies in living cells by modulating the DEP force. This study demonstrated that living cells can have non-linear di-polarized responses depending on the modulation direction of the applied frequency as well as providing a simple and reliable platform from which to measure a cellular cross-over frequency and characterize its nonlinear property.

## 1. Introduction

When a living cell is exposed to an alternating current (AC) electric field, dipoles are created inside the cell. The interaction between the dipoles and the AC electric field generates force, and that force moves the di-polarized cell. This phenomenon is called dielectrophoresis. The orientation of cell behavior as a function of applied field is controlled by the real value of the Clausius-Mossotti factor, which relates to the frequency-dependent dielectric polarization of cells in relation to the surrounding medium [1]. For example, at low AC frequency, MCF-7 cell and *E. coli* bacteria cell move in the direction of the minimum electric field gradient because the cells are less polarized than the medium, while the reverse occurs at high AC frequency [2,3]. Thus, cellular behavior can be easily controlled by varying the frequency of the AC electric field. That simplicity has driven the widespread use of dielectrophoretic (DEP) force in the manipulation of living cells in diverse practical applications such as single cell 3D manipulation [4,5] cell sorting [6], cell transfer [7], cell separation [8,9], and electrolysis [10]. Furthermore, the DEP technique is increasingly being recognized as a potentially valuable tool for characterizing the electrophysiological properties of various cell lines (e.g., cancer cells [11], stem cells [12], and bacteria [13]), as well as cell response by apoptosis [14,15] and cell responses to chemical agents [16,17,18], medium conditions [19], and circadian rhythms [20]. In those applications, the identification of cell DEP behavior as a function of AC frequency provides useful information about electrophysiological parameters [1].

To date, many methods have been used to characterize cellular behavior with respect to AC frequency. The initial approach to characterizing cellular DEP behavior was to count the number of cells captured by the electrode in a microfluidic channel as the cells responded to a positive DEP force combined with a laminar flow [2,21,22]. However, that approach could not characterize cellular behavior in the low frequency region in which negative DEP force acts on cells. Alternatively, the reflected light intensity within a specific area around the electrode can be monitored using fluorescence microscopy [23,24,25] or optical microscopy [14,18,20] to study the cellular reactions to DEP force. Cells are trapped on the edge of the electrode or dispersed around the electrode as the DEP force changes as a function of frequency, resulting in observable changes in light intensity that can be used to characterize DEP behavior. Another option is a cell tracking technique using a pair of electrodes [26], a quadrupole [13], or an interdigitated electrode (IDT) [11,12,17,27] to estimate changes in cell velocity with frequency. Both the microscopy and tracking approaches have been used while applying the positive or negative DEP force needed for a specific task, and both techniques can measure a quantitatively distinct feature for characterizing cell behavior. Although the significant efforts made to date have provided key information about electrophysiological properties, no one has yet observed cellular behavior while the frequency of the applied input AC signal sequentially increased and decreased in a single condition. The frequency of the electrical input used in the above assays was always applied in monotonic rather than successive increases and decreases. Thus, previous research assumed that cellular responses to frequency changes would be linear and unrelated to the direction of variation. However, many reports have described nonlinear electrodynamic responses as a function of various external stimuli and internal cell signal processes [16,28,29,30,31,32]. Therefore, cellular DEP behaviors could vary if the input frequency is successively increased and decreased. In addition, those differences can help manipulate cellular behaviors more precisely, as well as enabling a better method to characterize electrophysiological properties.

For this paper, we fabricated an IDT with circular windows to easily observe cellular behaviors while a modulating DEP force as sequentially increasing and decreasing frequencies or vice versa, and we developed a measurement method to simultaneously trace the behaviors of many MCF-7 cells with single-cell level resolution. Using the developed method, we observed traces of more than 150 MCF-7 cells responding to DEP forces inside the IDT electrode at the same time, identifying a nonlinear dynamic property (i.e., hysteresis loop) during modulation of the DEP force. In addition, we simultaneously measured the effective cross-over frequencies of numerous cells with single-cell level condition using the developed method and found different cross-over frequencies caused by the nonlinear cellular DEP behavior, even though the measurements is were performed in the same environment.

## 2. Materials and Methods

### 2.1. Chip Design and Fabrication

Our DEP microfluidic (DEP-μF) chip was fabricated on an oxide/silicon wafer (i-Nexus, Seongnam, Korea) substrate using a standard microfabrication technique [16]. Briefly, the first step in chip fabrication was the production of microelectrodes (i.e., interdigitated electrode array (30 μm wide and 10 μm separation)) through the lift-off process, followed by the formation of an insulator layer (8000-Å thick silicon dioxide) deposited using the plasma-enhanced chemical vapor deposition (PECVD) process. Subsequently, the contact pad portions and the circle windows on the IDT array were etched with buffered hydrofluoric acid solution (J.T. Baker Chemical Co, Phillipsburg, NJ, USA). To observe the behavior of living cells on the DEP-μF chip, a 1.2-mm-thick polydimethylsiloxane (PDMS) well was put into contact with the IDT array chip surface. The detailed process of chip fabrication is shown in Supplementary Materials S-1.

### 2.2. Cell Culture and Viability

**Figure 1:**
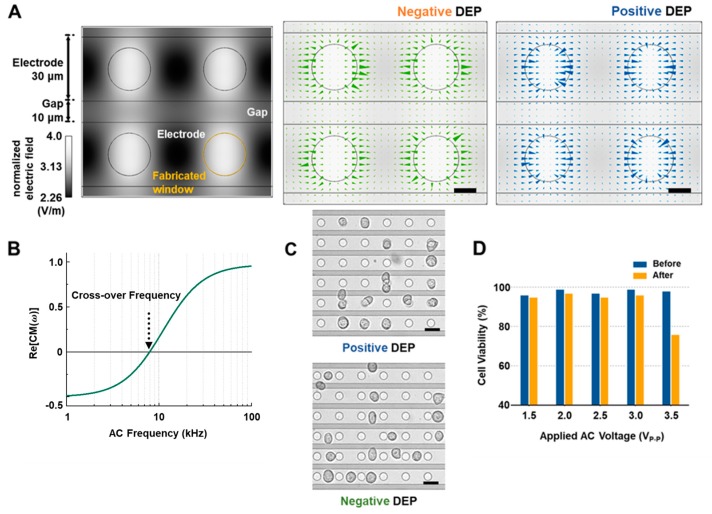
(**A**) Finite Element Method (FEM)-based simulation of the induced electric field distribution and positive and negative DEP force vectors pointing toward the corresponding trap region at the 9.8 μm height that was located on the center of MCF-7 cell. Scale bar, 10 μm. (**B**) The simulated real part of the Clausius-Mossotti factor, Re[CM], as a function of input frequency. (**C**) Optical images of biological cells moving in response to positive and negative DEP forces from an applied current of 2 V_p-p_ at 41 kHz and 1 kHz, respectively, where the average radius of MCF-7 and the step height between the inside window and the outside window are 9 μm and 0.8 μm (see Supplementary Materials S-1 and S-5). Scale bar, 30 μm. (**D**) Trypan blue cell viability assay to test DEP operation.

Human breast-cancer cell line MCF-7 cells were grown in DMEM (Dulbecco’s modified Eagle’s medium, Lonza, Walkersville, MD, USA) containing 10% FBS (fetal bovine serum, Gibco, Gaithersburg, MD, USA) and 1% penicillin-streptomycin (Gibco). The cell culture was incubated at 37.5 °C in a humidified atmosphere of 5% CO_2_. Before microfluidic cell experiments were performed inside our DEP-μF chip, the cells were detached by trypsin/EDTA, washed three times, and transferred to DEP experiment buffer of 8.6% (w/w) sucrose, 0.3% (w/w) D-glucose, 0.20% (v/v) phosphate-buffered saline (PBS; ×1) (Gibco), and 1.0 mg/mL BSA (bovine serum albumin, Sigma-Aldrich, St. Louis, MO, USA) by centrifugation procedure [33], where the measured conductivity of the DEP experiment buffer was 60 μS/cm. In addition, cell viability was investigated using Trypan blue (Sigma-Aldrich) staining before and after microfluidic cell experiments were performed. MCF-7 cells on the DEP-μF chip were exposed for about 2-h to a variety of alternating current (AC) voltages (1.5, 2.0, 2.5, 3.0, and 3.5 V_peak-peak_). After exposure, the solution was mixed with 0.4% Trypan blue solution at a 1:1 ratio and the number of viable cells were counted using a hemocytometer (Paul Marienfeld GmbH & Co. KG, Lauda-Königshofen, Germany) equipped with an inverted microscope (BS2090, Bestscope, Beijing, China). The result of cell viability is in Figure 1D.

### 2.3. Modeling and Simulations of DEP Force Acting on MCF-7 Cells

The following model has been used for the simulation study. The DEP force is described by

(1) $$F_{DEP}=2\pi \varepsilon_{medi}r_{Cell}^{3}Re\left[CM\right]\nabla {\left|E_{RMS}\right|}^{2}$$

where *ε_medi_* is the medium permittivity, *r_cell_* is the radius of a cell, *CM* is the Clausius-Mossotti (CM) factor, and *E_RMS_* is the RMS electric field [34]. The CM factor model assumes that the cell adheres to the single shell model [1]. Hence, the CM factor is given by

(2) $$CM=\left(\varepsilon_{cell}^{*}-\varepsilon_{medi}^{*}\right)/\left(\varepsilon_{cell}^{*}+2\varepsilon_{medi}^{*}\right)$$

(3) $$\varepsilon_{cell}^{*}=\varepsilon_{memb}^{*}\frac{\left[\lambda^{3}+\left\{\left(\varepsilon_{cyto}^{*}-\varepsilon_{memb}^{*}\right)/\left(\varepsilon_{cyto}^{*}+\varepsilon_{memb}^{*}\right)\right\}\right]}{\left[\lambda^{3}-\left\{\left(\varepsilon_{cyto}^{*}-\varepsilon_{memb}^{*}\right)/\left(\varepsilon_{cyto}^{*}+\varepsilon_{memb}^{*}\right)\right\}\right]}$$

where ε_cell_*, ε_medi_*, ε_memb_*, and ε_cyto_* are the complex permittivities of the cell, the medium, cell membrane, and the cytoplasm and ε* = ε + *j*(*σ*/*ω*) where *ε*, *σ*, and *ω* are the relative permittivity, conductivity, and angular frequency, respectively. *λ* is the scaling of cells described as *λ = r*_cell_/(*r_cell_-t*), where *t* is the thickness of the cell membrane. To predict the trapping location of the MCF-7 cell during the experiment, we designed the DEP chip structure with the same dimensions as used in the experiment using finite-element software (COMSOL Multiphysics, 3.5a, more detailed information on the structure in Figure S2 (see Supplementary Materials)). When the AC signal with a certain frequency is applied it in the IDT array structure, the electric field was generated inside the structure (Figure S2). Associating the field data with Equations (1)–(3), the magnitude of directional DEP force acting on the MCF-7 cell and the crossover frequency of it were numerically simulated, as shown in Figure 1A,B, where, the measured value of the average radius of MCF-7 cells is 9.034 ± 0.2256 μm (Figure S5), and the parameters used in the simulation for the MCF-7 cell model are shown in Table S1 (see Supplementary Materials).

### 2.4. Experimental Configuration

The DEP-μF chip was first bathed in a piranha solution (H_2_O_2_:H_2_SO_4_ = 1:1) to clean organic residues off the substrate, and a PDMS reservoir was attached to the chip. Buffer solution including cells (100 cells/μL) was introduced into the reservoir. The top of the reservoir was sealed by cover glass. An AC signal was applied to the contact pad on the chip using a custom probe station (Modusystems, Inc., Hanam, Korea) which connected to a LabVIEW (National Instruments, Austin, TX, USA)-based automated DEP system [35]. Numerous MCF-7 cells behaviors were observed and recorded under identical conditions using a charge-coupled device camera (Motionscope M3, Redlake, San Diego, CA, USA). The detailed DEP set-up is shown in Figure S3.

### 2.5. Method for Observing Cellular Behavior Using DEP Tweezers in a Microfluidic Device

Accurate quantification of cellular behavior is critically important for the characterization of non-linear dielectrophoretic cellular behavior. To quantify the behavior, we developed two methods. First, we followed a cell behavior tracking method. (1) We used the radial symmetry method [36] and the image processing method (MATLAB R17, Mathworks, Natick, MA, USA) to develop code to identify the centers of numerous cells in images recording sequential cellular behaviors (The detailed code development is explained in Supplementary Materials S-4); (2) By combining the code with images of cells trapped by DEP force at different positions, we determined the center of each cell; (3) By repeating the second step for 1000 frames while applying constant DEP force to each cell, we obtained 1000 center positions for each cell; (4) Using those center data for each cell, we calculated statistically reliable distribution functions corresponding to each cell, resulting in the average (m) center position with a standard deviation (*σ*) for each cell; (5) We were then able to define the center position as a reference point for each cell; (6) The criterion for judging the behavior of each cell is thus the moment of the reference point greater than 2*σ* as the DEP force acting on the cell changes.

For the second approach, we modified the grayscale measurement method used to evaluate the time-dependent grayscale values corresponding to the upward behavior of micro-objects [37,38] to measure the lateral behavior of a cell, as follows. (1) During the experiment, we defined two different circular windows in the chip. One window is the fabricated real window, and the other is an artificial window localized on the center of a cell (see Figure 3A,B). To automatically detect the many defined circular windows in the image, the center position of each circular window in the entire region is found by applying the image processing method for the fabricated window and the cell tracking method for the artificial circular window; (2) Using these data and the grayscale measurement method described in previous reports, we evaluated the time-dependent grayscale values within each window as a cell was ejected from an artificial window or injected into a fabricated window as the DEP force changed; (3) Then, we generated a statistically reliable distribution function with *μ* and *σ* using those grayscale values; (4) The criterion for judging complete injection into the fabricated window and ejection from the artificial windows is that the grayscale value changes more than 2*σ* as the DEP force acting on the cell changes. The behaviors of MCF-7 cells in the DEP-μF chip were quantified using both those methods and the more detailed quantification analysis described in Supplementary Materials S-6.

## 3. Results and Discussions

### 3.1. Numerical Simulation, Cell Alignment and Viability Results

The simulation results of our interdigitated electrode with circular windows, where radius of the window, gap between electrodes, and width of the electrode are 10 µm, 10 µm, and 30 µm, respectively, are shown in Figure 1A,B. According to Figure 1B, the cross-over frequency is about 7.9 kHz when the following parameters are applied: the permittivities and conductivities of MCF-7 and the buffer solution are 50 *ε*_0_(*ε*_cyto_), 80 *ε*_0_(*ε_m_*_emb_), 78.5 *ε*_0_(*ε*_medi_), 0.01 S/cm, and 60 μS/cm, respectively (Table S1) [3]. Below the cross-over frequency, negative DEP force pushes the cell to in-between the windows, where the electric field gradient is minimized. Above the cross-over frequency, the cell moves into the window where the electric field gradient is maximized under positive DEP force, resulting in the alignment of MCF-7 cells shown in Figure 1C. Figure 1D shows cell viability percentages before and after applying AC signals under the same conditions that we would use for the observation of cellular behavior in the fabricated dielectrophoresis microfluidic (DEP-μF) chip. According to these results, the viability of MCF-7 cells is above 95% before and after applying the experimental conditions when the applied voltage is below 3.0 V_p-p_. Hence, any voltage below 3.0 V_p-p_ might be selected for the experiment. However, we selected the voltage by the following criteria: the voltage should be as small as possible, and the voltage should be sufficient to move the cell. Under these criteria, 2.0 V_p-p_ was selected for further investigation.

### 3.2. Cellular Behaviors Characterization near Windows

**Figure 2:**
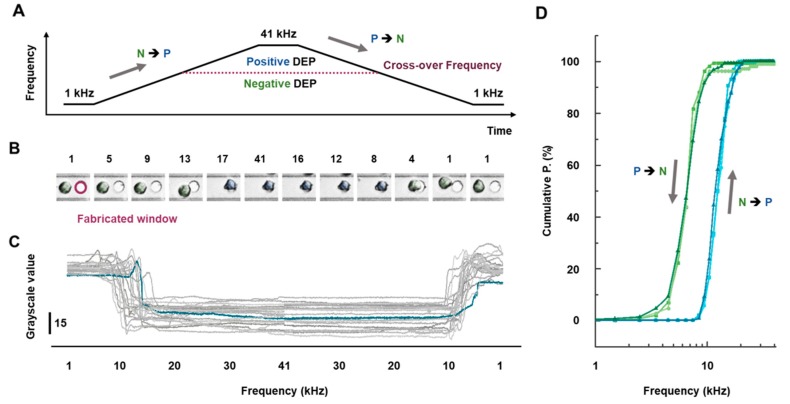
(**A**) Schematic illustration of the input AC frequency waveform in response to different modulation directions (i.e., 1 → 41 → 1 kHz in 2V_p-p_), where the frequency modulation rate was 0.4 kHz/s (**B**) In situ monitoring of cellular DEP behaviors, showing an individual cell trapped in the positive and negative trap regions during increasing and decreasing frequency modulations. (**C**) Variation of grayscale levels in the circular window region as AC frequency changed (1 → 41 → 1 kHz). The blue line is the grayscale value in response to the cellular behavior shown in the sequential images in Figure 2B. The AC input frequency was fixed for 100 s each at 1 and 41 kHz to stabilize the trapped cell motion. (**D**) Hysteresis curve of the cumulative percentage as a function of the frequency for cells stopping just inside the window regions after entering and the trapped cells inside the windows started to move during the frequency modulation period shown in Figure 2A.

Cellular behaviors were observed while the frequency of the applied AC signal increased from 1 kHz to 41 kHz, and while it decreased back down to 1 kHz (Figure 2A). The sequential images of the cell behaviors near the trap window in Figure 2B are shown with the modulation of the frequency. The DEP force acting on the cell shifted from the negative into the positive regions as the frequency increased from 1 to 41 kHz and from positive to negative as the frequency decreased, as shown in Figure 2B. To better quantify individual cellular behavior, the two methods described in Materials and Methods were applied to the sequential images shown in Figure 2B. As a result, the grayscale variation in the fabricated windows corresponds to cellular behaviors in the sequential images, as shown by the blue line in Figure 2C. The grayscale variation in Figure 2C can be interpreted as follows: the values are highest when no cell is inside the fabricated window (e.g., 1st, 2nd, and 3rd images in Figure 2B) and begin decreasing as a cell moves into the window (e.g., 4th and 5th images in Figure 2B). The values converge at the minimum after a cell fills the window (e.g., 6th, 7th, 8th, and 9th images in Figure 2B). The grayscale value should then increase again as the cell moves out of the window (e.g., 10th image in Figure 2B), again reaching the maximum value when the cell has completely exited the window region (e.g., 11th and 12th images in Figure 2B). Using this system, we simultaneously observed more than 150 cells inside the microfluidic chip under the same experimental conditions. Their individual trace results during frequency modulation are shown in Figure 2C. From these data, we extracted the commutative percentage, which is that cells stopped just inside the window regions after entering while the frequency increased from 1 KHz to 41 KHz (i.e., N → P in Figure 2A) and the trapped cells inside the windows started to move while the frequency decreased from 41 KHz to 1 KHz (i.e., P → N in Figure 2A), as a function of frequency, resulting in the hysteresis curve shown in Figure 2D. The blue and green curves occur as the DEP force shifts from negative to positive and vice versa, respectively. Moreover, the cellular behaviors observed in our DEP-μF chip can be considered to result only from the effects of the DEP force acting on the cells. The electro-hydrodynamic effect generated by the applied AC input can be ignored because the input signal has the low amplitude and low frequency (2 V_p-p_ with 1–41 kHz) and the IDT array is insulated except at the fabricated windows [23,39,40]. Moreover, the cell-size distribution can also be ignored for construction of hysteresis curves for the reasons given in Supplementary Materials S-5. Therefore, this phenomenon offers clear evidence that MCF-7 cells have a nonlinear dynamic response to DEP force modulating in different directions.

### 3.3. Cellular Cross-Over Frequency Characterization

**Figure 3:**
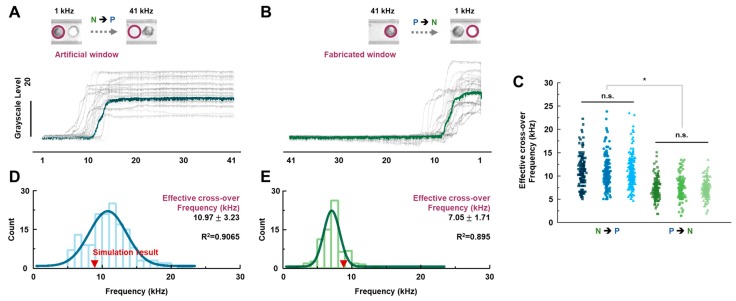
(**A**,**B**) Variation in grayscale levels in the circular trap region of the cell affected by negative and positive DEP forces in accordance with changes in frequency (1 → 41→ 1 kHz). (**C**) Effective cross-over frequencies of the biological cells under increasing and decreasing frequency modulations when the experiment was repeated in triplicate (* *p* < 0.001 and “n.s” represent “non-significant” (*p* > 0.5)). (**D**,**E**) Histogram and Gaussian fit of the effective cross-over frequencies measured during increasing and decreasing frequency modulations, respectively. The simulation point is the cross-over frequency, shown in Figure 1B.

To better understand the effects of those nonlinear cellular DEP behaviors, we characterized the cross-over frequencies (ideally the point at which $F_{DEP}$ becomes zero) during the different modulations. Because it is not possible to observe cellular behavior at the ideal cross-over frequency, we defined and measured the effective cross-over frequency (i.e., $F_{DEP}\approx 0$) as follows. We applied the cell tracking method to an individual cell trapped by negative DEP force and found its center position, on which we defined an artificial window (see the inset in Figure 3A). The blue line in Figure 3A represents the measured grayscale level of the artificial window described in the inset of Figure 3A. According to the blue line, the grayscale value stayed at the same level when the cell was in the artificial window because of the negative DEP force acting on it (1 kHz to 10.2 kHz in Figure 3A). The grayscale level started to increase when that cell started to move out of the window at a certain frequency (10.6 kHz in Figure 3A), indicating that the polarization of the DEP force changed. We defined that frequency as the effective cross-over frequency for an N → P modulation. For a P → N modulation, on the other hand, the cell was first located inside the fabrication window, during which the grayscale level stayed at the minimum value, as shown in Figure 3B. The grayscale level started to increase just as the cell started to move as a result of the positive DEP force changing to a negative DEP force. We define that frequency as the effective cross-over frequency for a P → N modulation. It should be noted that the effective frequency is not perfectly matched to the ideal cross-over frequency because the force is not zero at the effective cross-over frequency. However, the measured effective cross-over frequency is the best approximation to the ideal value in our measurement system, which has 414 nm (2*σ*) resolution for detecting cellular behavior (more detailed information is in Supplementary Materials S-6). The effective cross-over frequencies of more than 150 cells were measured while applying different modulation directions to the DEP force, resulting in the finding illustrated in Figure 3D,E. Distinct Gaussian distributions are shown, with statistically reliable N → P and P → N cross-over frequencies of 10.97 ± 3.23 kHz and 7.05 ± 1.71 kHz, respectively. The experiments were repeated three times under the same conditions. Figure 3C shows the results, indicating that the effective cross-over frequencies are not statistically different for the same modulation direction in DEP force, but they are distinguishable for different modulation directions in DEP force.

This nonlinear dynamic response represented by the hysteresis curve and different cross-over frequencies during modulation of DEP force could explain the di-polarized tendency between a cell and the medium surrounding it. At 1 kHz, at which negative DEP force is maximized in the experiment, the electric dipoles in the medium mainly generate the force. As the frequency increases but remains below the cross-over frequency, the electric dipoles in the medium decrease, and the electric dipoles in the cell increase. At the cross-over frequency, the net dipole of the medium and the cell is zero. As the frequency continues to increase, the electric dipoles in the cell become more and more dominant, resulting in an increase in positive DEP force. When the frequency decreases from 41 kHz to 1 kHz, the reverse process should occur [1,41]. This different di-polarization tendency between cells and medium might explain the occurrence of different cross-over frequencies when negative DEP force switches to positive DEP force (N → P) and vice versa (P → N). Moreover, cells are heterogeneous, consisting of protein, DNA, a lipid bilayer, carbohydrates, and various organelles [42]. The heterogeneous materials of each cell can cause different di-polarized effects as frequency varies [43] and could also explain the nonlinear dynamic response reported in this paper. Another consideration in investigating the nonlinear dynamic response is the effect of frequency modulation rate because it has been reported that the modulation rate can affect cellular DEP behavior [44]. Since the modulation rate can perturb the dielectric polarization of cells, the nonlinear dynamic response in relation to the observed hysteresis could be affected. In these regards, further investigation should be needed for the better understanding of the non-linear dynamic behavior responded to the frequency modulation direction and rate. However, our new simple and robust measurement method has made it possible to observe this nonlinear cellular DEP behavior and different cellular cross-over frequencies that occur as DEP force modulates in different directions.

## 4. Conclusions

To measure the cross-over frequency and evaluate the nonlinear dynamics generated when a cell is di-polarized under an AC electric field, we developed a novel methodology using DEP tweezers in the microfluidic device. Through that innovation, we simultaneously observed the behaviors of many MCF-7 cells in the same environment as we modulated the DEP force in different directions. From the measurement data, we characterized a hysteresis curve corresponding to cellular behavior, and based on the nonlinear dynamic response, we precisely measured and examined the effective cross-over frequencies of cells in the system. We found distinct cross-over frequencies for MCF-7 cells for the two different modulation directions of DEP force, suggesting that cellular DEP behavior depends on the direction of frequency modulations. In this study, the developed method can be a very useful tool for measuring cellular cross-over frequency and evaluating nonlinear dynamic responses of di-polarized cells. Also, the nonlinear dynamic responses described in this report provide important information for understanding the dielectric properties of living cells.

## Supplementary Materials

All data to support the main article are available at http://www.mdpi.com/1424-8220/18/10/3543/s1.

## References

[1] Pethig R. (2017). Dielectrophoresis: Theory, Methodology and Biological Applications.

[2] Coley H.M., Labeed F.H., Thomas H., Hughes M.P. (2007). Biophysical characterization of MDR breast cancer cell lines reveals the cytoplasm is critical in determining drug sensitivity. Biochim. Biophys. Acta-Gen. Subj..

[3] Henslee E.A., Sano M.B., Rojas A.D., Schmelz E.M., Davalos R.V. (2011). Selective concentration of human cancer cells using contactless dielectrophoresis. Electrophoresis.

[4] Kung Y.-C., Huang K.-W., Fan Y.-J., Chiou P.-Y. (2015). Fabrication of 3D high aspect ratio PDMS microfluidic networks with a hybrid stamp. Lab Chip.

[5] Kung Y.C., Huang K.W., Chong W., Chiou P.Y. (2016). Tunnel Dielectrophoresis for Tunable, Single-Stream Cell Focusing in Physiological Buffers in High-Speed Microfluidic Flows. Small.

[6] Hu X., Bessette P.H., Qian J., Meinhart C.D., Daugherty P.S., Soh H.T. (2005). Marker-specific sorting of rare cells using dielectrophoresis. Proc. Natl. Acad. Sci. USA.

[7] Clow A.L., Gaynor P.T., Oback B.J. (2010). A novel micropit device integrates automated cell positioning by dielectrophoresis and nuclear transfer by electrofusion. Biomed. Microdevices.

[8] Moon H.-S., Kwon K., Kim S.-I., Han H., Sohn J., Lee S., Jung H.-I. (2011). Continuous separation of breast cancer cells from blood samples using multi-orifice flow fractionation (MOFF) and dielectrophoresis (DEP). Lab Chip.

[9] Faraghat S.A., Hoettges K.F., Steinbach M.K., van der Veen D.R., Brackenbury W.J., Henslee E.A., Labeed F.H., Hughes M.P. (2017). High-throughput, low-loss, low-cost, and label-free cell separation using electrophysiology-activated cell enrichment. Proc. Natl. Acad. Sci. USA.

[10] Jung Y.-J., Lee T., Choi S., Lee S.-Y., Key J., Yoo Y.-M., Chang W.-J., Kim H.-S., Lee S.W. (2017). Selective position of individual cells without lysis on a circular window array using dielectrophoresis in a microfluidic device. Microfluid. Nanofluid..

[11] Vaillire C., Honegger T., Kermarrec F., Gidrol X., Peyrade D. (2016). Label-Free Electric Monitoring of Human Cancer Cells as a Potential Diagnostic Tool. Anal. Chem..

[12] Velugotla S., Pells S., Mjoseng H.K., Duffy C.R.E., Smith S., De Sousa P., Pethig R. (2012). Dielectrophoresis based discrimination of human embryonic stem cells from differentiating derivatives. Biomicrofluidics.

[13] Elitas M., Dhar N., Schneider K., Valero A., Braschler T., McKinney J.D., Renaud P. (2017). Dielectrophoresis as a single cell characterization method for bacteria. Biomed. Phys. Eng. Express.

[14] Mulhall H.J., Cardnell A., Hoettges K.F., Labeed F.H., Hughes M.P. (2015). Apoptosis progression studied using parallel dielectrophoresis electrophysiological analysis and flow cytometry. Integr. Biol..

[15] Henslee E.A., Torcal Serrano R.M., Labeed F.H., Jabr R.I., Fry C.H., Hughes M.P., Hoettges K.F. (2016). Accurate quantification of apoptosis progression and toxicity using a dielectrophoretic approach. Analyst.

[16] Park I.S., Lee J., Lee G., Nam K., Lee T., Chang W.-J., Kim H., Lee S.-Y., Seo J., Yoon D.S. (2015). Real-Time Analysis of Cellular Response to Small-Molecule Drugs within a Microfluidic Dielectrophoresis Device. Anal. Chem..

[17] Vaillier C., Honegger T., Kermarrec F., Gidrol X., Peyrade D. (2015). Involvement of membrane proteins and ion channels on the self-rotation of human cells in a non-rotating AC electric field. Electrophoresis.

[18] Hoettges K.F., Hübner Y., Broche L.M., Ogin S.L., Kass G.E.N., Hughes M.P. (2008). Dielectrophoresis-Activated Multiwell Plate for Label-Free High-Throughput Drug Assessment. Anal. Chem..

[19] Chung C., Pethig R., Smith S., Waterfall M. (2017). Intracellular potassium under osmotic stress determines the dielectrophoresis cross-over frequency of murine myeloma cells in the MHz range. Electrophoresis.

[20] Henslee E.A., Crosby P., Kitcatt S.J., Parry J.S.W., Bernardini A., Abdallat R.G., Braun G., Fatoyinbo H.O., Harrison E.J., Edgar R.S. (2017). Rhythmic potassium transport regulates the circadian clock in human red blood cells. Nat. Commun..

[21] Broche L.M., Bhadal N., Lewis M.P., Porter S., Hughes M.P., Labeed F.H. (2007). Early detection of oral cancer—Is dielectrophoresis the answer?. Oral Oncol..

[22] Labeed F.H., Coley H.M., Thomas H., Hughes M.P. (2003). Assessment of Multidrug Resistance Reversal Using Dielectrophoresis and Flow Cytometry. Biophys. J..

[23] Zellner P., Shake T., Sahari A., Behkam B., Agah M. (2013). Off-chip passivated-electrode, insulator-based dielectrophoresis (OπDEP). Anal. Bioanal. Chem..

[24] Shake T., Zellner P., Sahari A., Breazeal M.V.R., Behkam B., Pruden A., Agah M. (2013). Embedded passivated-electrode insulator-based dielectrophoresis (EπDEP). Anal. Bioanal. Chem..

[25] Nakidde D., Zellner P., Alemi M.M., Shake T., Hosseini Y., Riquelme M.V., Pruden A., Agah M. (2015). Three dimensional passivated-electrode insulator-based dielectrophoresis. Biomicrofluidics.

[26] Lo Y.J., Lin Y.Y., Lei U., Wu M.S., Yang P.C. (2014). Measurement of the Clausius-Mossotti factor of generalized dielectrophoresis. Appl. Phys. Lett..

[27] Su H.-W., Prieto J.L., Voldman J. (2013). Rapid dielectrophoretic characterization of single cells using the dielectrophoretic spring. Lab Chip.

[28] Kirkegaard J., Clausen C., Rodriguez-Trujillo R., Svendsen W. (2014). Study of Paclitaxel-Treated HeLa Cells by Differential Electrical Impedance Flow Cytometry. Biosensors.

[29] Bagnaninchi P.O., Drummond N. (2011). Real-time label-free monitoring of adipose-derived stem cell differentiation with electric cell-substrate impedance sensing. Proc. Natl. Acad. Sci. USA.

[30] Reitinger S., Wissenwasser J., Kapferer W., Heer R., Lepperdinger G. (2012). Electric impedance sensing in cell-substrates for rapid and selective multipotential differentiation capacity monitoring of human mesenchymal stem cells. Biosens. Bioelectron..

[31] Asami K., Gheorghiu E., Yonezawa T. (1999). Real-Time Monitoring of Yeast Cell Division by Dielectric Spectroscopy. Biophys. J..

[32] Wang L., Wang L., Yin H., Xing W., Yu Z., Guo M., Cheng J. (2010). Real-time, label-free monitoring of the cell cycle with a cellular impedance sensing chip. Biosens. Bioelectron..

[33] Sabuncu A.C., Liu J.A., Beebe S.J., Beskok A. (2010). Dielectrophoretic separation of mouse melanoma clones. Biomicrofluidics.

[34] Pohl H.A. (1978). Dielectrophoresis—The Behavior of Neutral Matter in Nonuniform Electric Fields.

[35] Kim M.H., Lee J., Nam K., Park I.S., Son M., Ko H., Lee S., Yoon D.S., Chang W.-J., Lee S.Y. (2017). Automated Dielectrophoretic Tweezers-Based Force Spectroscopy System in a Microfluidic Device. Sensors.

[36] Parthasarathy R. (2012). Rapid, accurate particle tracking by calculation of radial symmetry centers. Nat. Methods.

[37] Park I.S., Eom K., Son J., Chang W.J., Park K., Kwon T., Yoon D.S., Bashir R., Lee S.W. (2012). Microfluidic multifunctional probe array dielectrophoretic force spectroscopy with wide loading rates. ACS Nano.

[38] Son M., Choi S., Ko K.H., Kim M.H., Lee S.-Y., Key J., Yoon Y.-R., Park I.S., Lee S.W. (2016). Characterization of the Stiffness of Multiple Particles Trapped by Dielectrophoretic Tweezers in a Microfluidic Device. Langmuir.

[39] Pethig R. (2017). Where is dielectrophoresis (DEP) going?. J. Electrochem. Soc..

[40] Lee T.W., Nam K., Baek S.H., Chang W.J., Kim S.K., Kim H.S., Yoon D.S., Lee S.W. (2010). Numerical and Experimental Study on Dielectrophoretic and Electrohydrodynamic Traps using Micro-Particles on An Interdigitated Electrode Array System. Int. J. Nonlinear Sci. Numer. Simul..

[41] Valero A., Braschler T., Renaud P. (2010). A unified approach to dielectric single cell analysis: Impedance and dielectrophoretic force spectroscopy. Lab Chip.

[42] Raicu V., Feldman Y. (2015). Dielectric Relaxation in Biological Systems: Physical Principles, Methods, and Applications.

[43] Pethig R., Menachery A., Pells S., De Sousa P. (2010). Dielectrophoresis: A Review of Applications for Stem Cell Research. J. Biomed. Biotechnol..

[44] Adams T.N.G., Leonard K.M., Minerick A.R. (2013). Frequency sweep rate dependence on the dielectrophoretic response of polystyrene beads and red blood cells. Biomicrofluidics.

