# Supplementary material for: Non-Linear Cellular Dielectrophoretic Behavior Characterization Using Dielectrophoretic Tweezers-Based Force Spectroscopy inside a Microfluidic Device
Source: Sensors (Basel). 2018 Oct 19;18(10):3543. doi: 10.3390/s18103543 (PMC6210972; doi:10.3390/s18103543)
Supplement: Supplementary file 1 [file sensors-18-03543-s001.pdf]

sensors

MDPI

Article

Non-Linear Cellular Dielectrophoretic Behavior Characterization Using Dielectrophoretic Tweezers-Based Force Spectroscopy inside a Microfluidic Device

Seungyeop Choi [1,†], Kwanhwi Ko [1,†], Jongwon Lim [1], Sung Hoon Kim [2], Sung-Hun Woo [2], Yoon Suk Kim [2], Jaehong Key [1], Sei Young Lee [1], In Su Park [1,3,*] and Sang Woo Lee [1,*]

[1] Department of Biomedical Engineering, Yonsei University, Wonju 26493, Korea; sychoi0091@gmail.com (S.C.); ko.kwanhwi@gmail.com (K.K.); jongwonlim44@naver.com (J.L.); jkey@yonsei.ac.kr (J.K.); syl235@yonsei.ac.kr (S.Y.L.)
[2] Department of Biomedical Laboratory Science, Yonsei University, Wonju 26493, Korea; k140017@naver.com (S.H.K.); sunghun2120@gmail.com (S.-H.W.); yoonsukkim@yonsei.ac.kr (Y.S.K.)
[3] Micro and Nanotechnology Laboratory, University of Illinois at Urbana–Champaign, Urbana, IL 61801, USA
* Correspondence: insu1023@gmail.com (I.S.P.); yusuklee@yonsei.ac.kr (S.W.L.); Tel.: +82-33-760-2791 (S.W.L.)
† Seungyeop Choi and Kwanhwi Ko are equally contributed in this article.

Received: 3 September 2018; Accepted: 17 October 2018; Published: 19 October 2018

S-1. DEP Chip design and fabrication

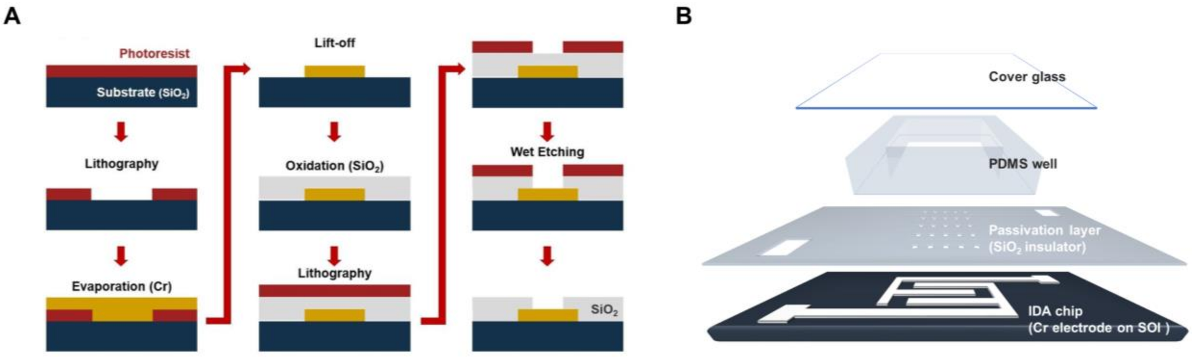

Figure S1. Fabrication of dielectrophoresis microfluidic (DEP-μF) chip: (**A**) insulated IDT array chip fabrication process; (**B**) the structure of the DEP-μF chip.

(**A**) **Insulated IDT array chip fabrication.** Insulated IDT array chip fabrication is the first step of DEP-μF chip fabrication (Figure S1-a). First, solvent cleaning of the bare SOI wafer was performed. The first wash was an acetone and methanol solution bath, followed by deionized water to clean off all chemical substances, and finally nitrogen was blown to dry the wafer. We also cleaned the chip by piranha solution (H_2O_2:H_2SO_4 = 3:1). After drying, the cleaned wafer was coated by the positive photoresist AZ GXR-601 (AZ electronic materials) using a spin coating machine. The spin coater was first adjusted to 300 rpm for 15 s to homogeneously spread the photoresist, then to 3000 rpm for 30 s to level the photoresist thickness to approximately 500 nm. Soft-baking was then carried out at 65 °C for 35 s using a hot plate. By using a photo-mask, the pattern was transferred to the wafer by exposure to UV light. The photoresist on the wafer was then developed by soaking in AZ 300 MIF developer (AZ electronic materials) for 10 s. After pattern inspection under a microscope, a 1000 Å thick chromium layer was deposited on the photoresist patterned substrate using the thermal evaporator. The rest of the photoresist was removed by acetone. Subsequently, the metal electrodes were covered by an 8000 Å thick layer of plasma-enhanced chemical vapor deposited (PECVD) silicon dioxide.

Finally, the insulated IDT array chip was partially etched for two electrode pads to connect with the electrode tip of a micro-positioner manipulator and for a 10-μm radius circle on the electrode. Insulated IDT array chips were thus produced.

(**B**) **DEP-μF chip fabrication.** To observe cell motion in buffer solution, we prepared a polydimethylsiloxane (PDMS) well. A 1.2-mm-thick PDMS plate was fabricated by degassing and curing. First, PDMS and prepolymer were mixed for 10 min (the mixture consisted of Sylgard 184, and Silicone Elastomer Sylgard 184 Curing Agent, 10:1 by volume). The mixture degassed in a vacuum chamber for 1 hour and cured in an oven at 65 °C for 3 hours. The PDMS well was made by custom square-stamp (6 × 6 × 1.2 mm^3) with a 3 × 3 × 1.2 mm^3-reservior and was cleaned in methanol solution during ultrasonication. Finally, the clean PDMS well was put into contact with the DEP chip surface.

S-2. Numerical analysis of DEP force acting on the cell

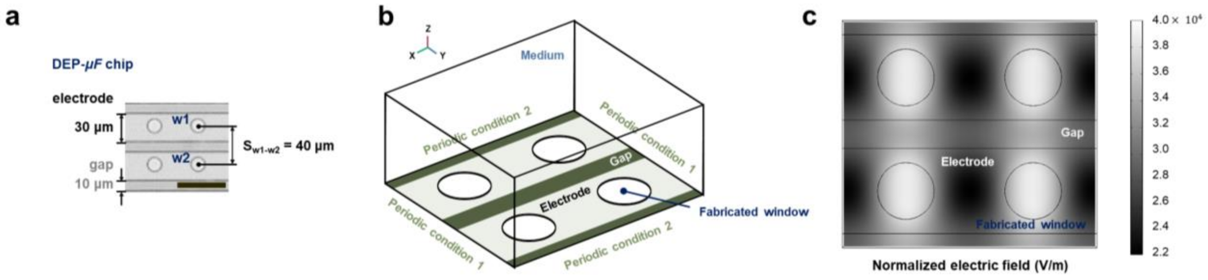

Figure S2. Finite-element simulation of the DEP-μF chip. (**a**) The DEP-μF chip is an electrode array 30 μm wide with a 10-μm gap, and the distance between the centers of the two circular windows is 40 μm. The scale bar is 50 μm. The IDT array structure is symmetric and can be spanned by the unit cell presented in (a). Therefore, the simulated results using (a) are adequate to cover the simulation results of the entire IDT array structure. (**b**) 3-Dimensional view of the DEP chip structure. (**c**) Simulation of the induced electric field distribution produced by the two-electrode structure.

Table S1. The dielectric properties of the MCF-7 cell. [1].

Cell Type	Average Radius (μm)	Permittivity ε (ε_0) Cytoplasm	Conductivity σ (S/cm) Cytoplasm	Capacitance (mF/m^2) Membrane	Cross-Over Frequency (kHz) Calculation
MCF-7	9.034	50	0.01	15-30	7.94

S-3. The setup of the μF-DEP system

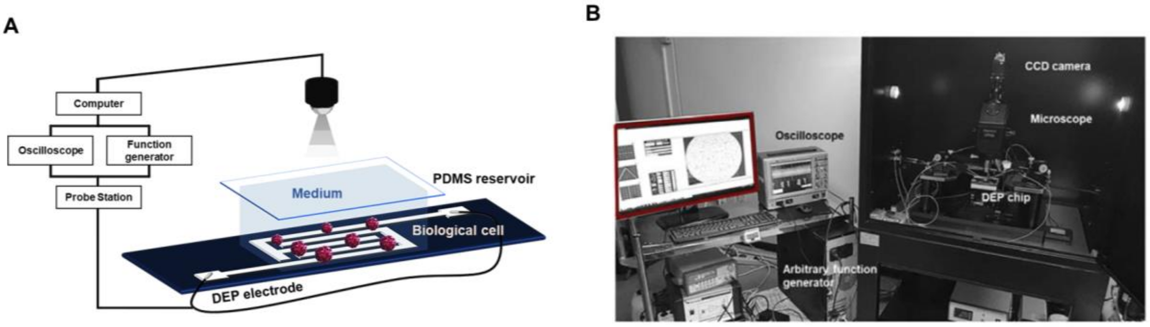

Figure S3. The setup of the μF-DEP measurement system. **(A)** Schematic illustration of the measurement process by DEP chip. **(B)** Real image of the DEP chip measurement device.

S-4. Programmable methods for measuring cell overlap with a fabricated window

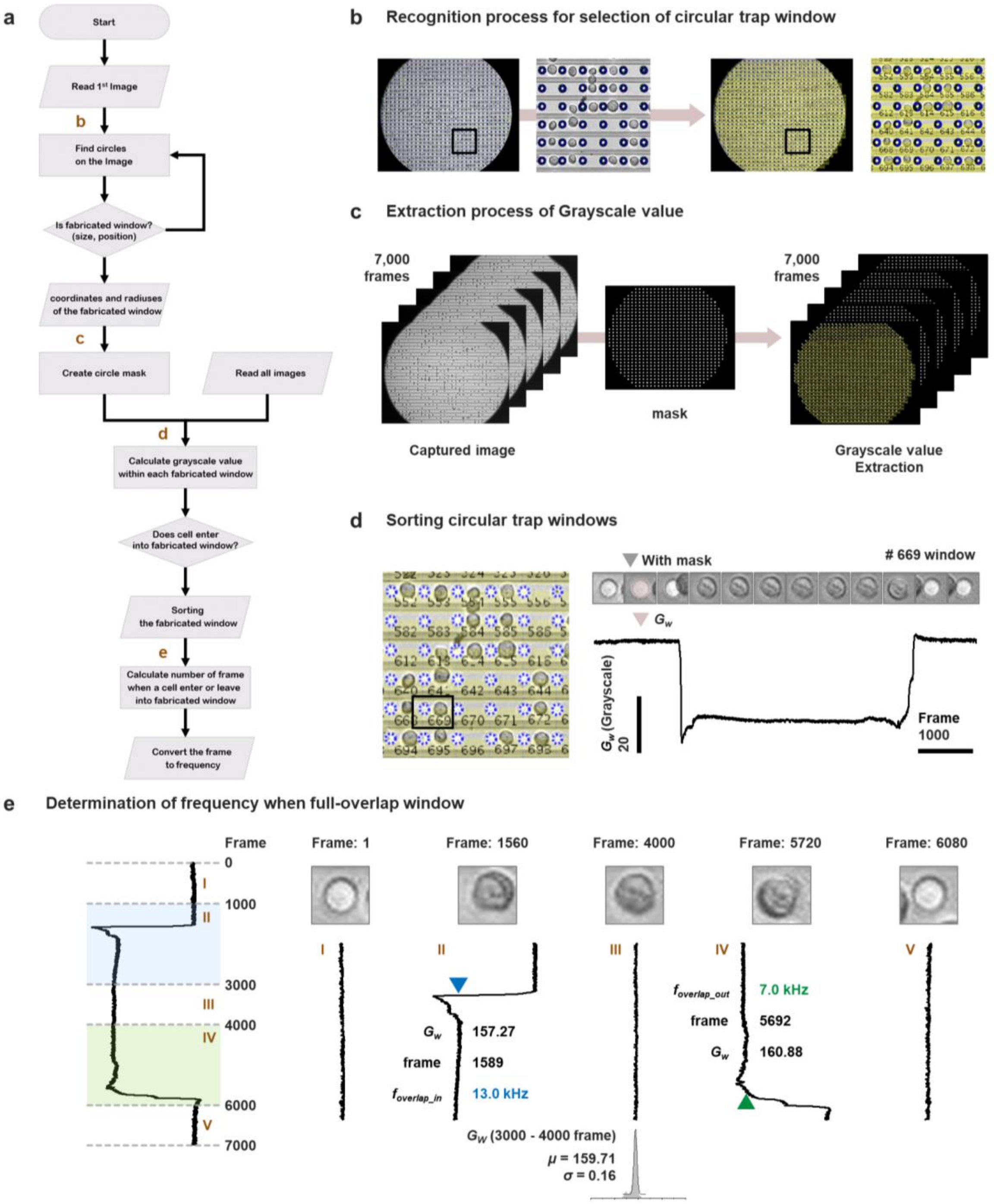

Figure S4. (**a**) Flowchart of the image measurement protocol. (**b**) Representative images of the entire field of view, the regions of interest centered on each circular window (dark blue circles), and magnified regions around each window. (**c**) Schematic illustration of the binary-mask application to extract grayscale intensity values (G_w) from the images (see text for details). (**d**) Variation of a representative overlap between a window and a cell and G_w corresponding to the variation during a frequency sweep. (**e**) Determination of the full-overlap frames and their conversion to frequency values from designated grayscale values.

In our system, the fabricated windows serve as effective motion detectors. We therefore used images to determine the extent to which a cell overlapped a given window. Those measurements enable realization of the frequency at which a cell stopped just inside the window regions after entering and the frequency at which a trapped cell started to move (i.e., $f_{overlap\_in}$ for 1–41 kHz, $f_{overlap\_out}$

for 41–1 kHz) by associating G_w with cell displacement. Consider, for example, an MCF-7 cell at a negative-DEP trap center in a 1 kHz field (see the first image in Figure 2B). Increasing the input signal frequency to 41 kHz changes the dielectric configuration between the cell and the medium, thereby shifting the equilibrium position of the cell from the negative trap region toward the fabricated window (see Figure 2B). In that process, G_w decreases gradually as the cell progressively overlaps the fabricated window, allowing the cell displacement to be determined as a function of the input frequency. The flowchart in Figure S4a describes the algorithm used for this calculation.

To interpret the variation in G_w, the location and radius of each fabricated window are also required. The dark blue circles in Figure S4b define a region of interest in the optical image that consists exclusively of the fabricated windows on the DEP chip. We developed MATLAB code based on the Circle Hough Transform method provided in the image processing toolbox (Mathworks, Natick, MA, USA) to select the fabricated windows in the images [2–4]. Because those fabricated windows have a well-defined radius (10 μm) and spacing (40 μm vertically and horizontally), they can be recognized as distinct circles (i.e., excluding cells and debris). Based on this geometry, a binary window mask was generated with "1" corresponding to a window region and "0" to the outside (Figure S4c). G_w values were then computed by multiplying this mask image by the measured time-lapse images and calculating the resulting image intensity over a sequence of 7000 frames (Figure S4d). The specific fabricated window group (all windows labeled with "number" in Figure S4d) included all fabricated windows with different grayscale values between the vacant window under the negative DEP trap and the full window under the positive DEP trap. The right-side panel in Figure S4d depicts a measurement artifact in a representative window (#669), where the G_w value was impaired by an irregularity in the etched surface. Figure S4e illustrates the method by which the frequency of full-overlap was determined using G_w.

S-5. The effect of cell size on nonlinear dynamic response

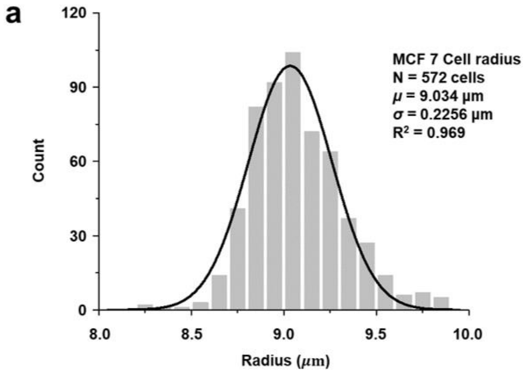

Figure S5. Measured radius of MCF-7 cells. The distribution was fitted by a Gaussian function.

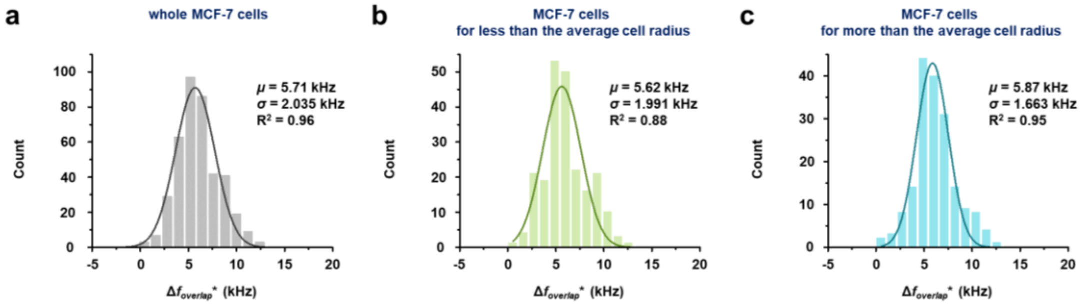

Figure S6. Histograms of discrepancy values as differences in cell size: (**a**) histogram of all cells, (**b**) histogram of cells that have a smaller-than-average cell radius, (**c**) histogram of cells that have a greater-than-average cell radius (for the average cell radius, see Figure S4a). $\Delta f_{overlap}$* is the differential frequency between $f_{overlap\_in}$ and $f_{overlap\_out}$ (see text in S-4 for details). A two-sample, assuming unequal variance t-test showed no differences among a, b, and c ($p > 0.1$).

The hysteresis curve shown in Figure 2D was produced from the cellular movement and the DEP force leads to cellular movement. Because the DEP force is proportional to the cubic power of the cell radius, there is possibility that distribution in cell size could affected the hysteresis curve (see Materials and Method in the main article). Therefore, we carefully investigated the effect of the cell-size distribution. First, we estimated the average radius of the cells, resulting in 9.034 ± 0.2256 μm, with an R^2 value of 0.969 (Figure S5). Then, the difference between $f_{overlap\_in}$ for 1–41 kHz and $f_{overlap\_out}$ for 41–1 kHz was defined as $\Delta f_{overlap}$* (i.e., $\Delta f_{overlap}$* $= f_{overlap\_in} - f_{overlap\_out}$), where the hysteresis curve can be constructed as a function of $f_{overlap\_in}$ and $f_{overlap\_out}$ when the applied frequency is increased and decreased, respectively. In accordance with the cell radius, $\Delta f_{overlap}$* was classified as follows: 1) entire cells not related to cell radius, 2) the cells that have less than the average radius, 3) the cells that have greater than the average radius. According to Figure S6, the distributions of $\Delta f_{overlap}$* for varying cell sizes are not statistically different. Therefore, the effect of cell size can be ignored in constructing the hysteresis loop.

S-6. Quantification of cellular movement in the measurement system

To analyze the resolution of our measurement system, we applied the two methods described in S-6, cell tracking and modified grayscale measurement. First, we applied the cell tracking method to track the center positions of cells trapped by negative DEP force and obtained complicated trajectories from time-sequential cellular movement images, as shown in Figure S7. Those trajectories can be called *thermal noise fluctuations*, generated even when the cell was trapped. Now, we assume that the trajectory on each sequential image is an independent, identically distributed, random variable. Then, the distributions of many cellular trajectories tend toward a Gaussian distribution with a probability density function, according to the central limit theorem [5]. The Gaussian distribution of x and y trajectories is given in Figure S7. Therefore, the movement variation is $\sigma_d = \sqrt{\sigma_x^2 + \sigma_y^2}$ = 207 nm. On the other hand, the moving distance from the statistical center of the trapped cell in the nth frame is defined as $D_n = C_n - C_o = \sqrt[2]{(x_n - x_o)^2 + (y_n - y_o)^2}$, where C_n is the nth cell center in the nth frame, and x_n and y_n are the nth position of the cell in Cartesian coordinates. C_o is the statistical center of the trapped cell, i.e., the average trapped position during a DEP trap period (i.e., 100 seconds at 1 kHz and 41 kHz). x_o and y_o represent the statistical center position in Cartesian coordinates. The variation in distance moved from the *n-1* frame to the nth frame is $\Delta D_n = D_n - D_{n-1}$. Therefore, when $\Delta D_n > 2\sigma$, we can identify cellular movement with a 95.3% confidence interval. Because 2σ is 414 nm in our measurement system, the resolution for realizing cellular movement is about 400 nm within a 95.3 % confidence interval.

To determine the resolution using the modified grayscale measurement method, we extract the average center point of the cell found by the cell tracking method, as shown in Figure S8a. Then, the artificial circular window can be created using that average center point, as shown in Figure S8b. The grayscale level in the artificial circular window also oscillates with thermal energy, even when the cell is trapped by DEP force. Assuming the fluctuation of the grayscale level to be an independent identical random variable, the central limit theorem indicates that it should follow a Gaussian distribution. Figure S8b is the result. Applying the same argument used above in the modified grayscale measurement method, we define the cell as having moved when the grayscale value in the artificial circular window exceeds 2σ (1.15 grayscale level). That method also identifies cellular movement with a 95.3% confidence interval. However, this is a qualitative approach. To take a more analytical approach to the experimental grayscale measurement data, we developed a model in which $S_o = \pi R_o^2$ is the statistical cell surface area in the artificial window, where R_o is the statistical radius of a cell, as shown in Figure S8. Then, we define K_n as the nth out-of-area ratio originating from the mismatched region between S_o and the circular area with the moving distance in the nth frame image. The mismatched area is thus A_n, as shown in Figure S9a, and is described by

$$A_n = K_n S_o = \pi R_{cell}^2 - \frac{1}{2}\{R_o^2(\theta_o - sin\theta_o) - R_n^2(\theta_n - sin\theta_n)\} \quad \text{(S4)}$$

where θ_o and θ_n are the angles in the nth area of overlapping circles (i.e., statistically trapped cell position and cell moving position in the nth frame, respectively). θ_o and θ_n are refined using the 2nd law of cosines as

$$\theta_o = 2\cos^{-1}(\frac{D_n^2 - (R_o^2 - R_n^2)}{2R_oD_n}), \quad \theta_n = 2\cos^{-1}\left(\frac{D_n^2 + (R_o^2 - R_n^2)}{2R_oD_n}\right) \tag{S5}$$

Using this model, the area fluctuation is calculated, resulting in Figure S9b. From these data, cellular movement can be assumed when the off-set area is 3.28 μm² (2σ). Therefore, 1.3% area variation corresponds to 1.15 grayscale level (2σ) in our measurement system.

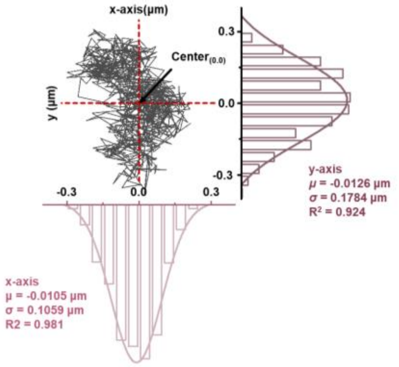

Figure S7. Characterization of cell trapping motion (AC 2 $V_{P\text{-}P}$, 1 kHz): Cell trajectory and cell location histogram are on the x-axis and y-axis, respectively.

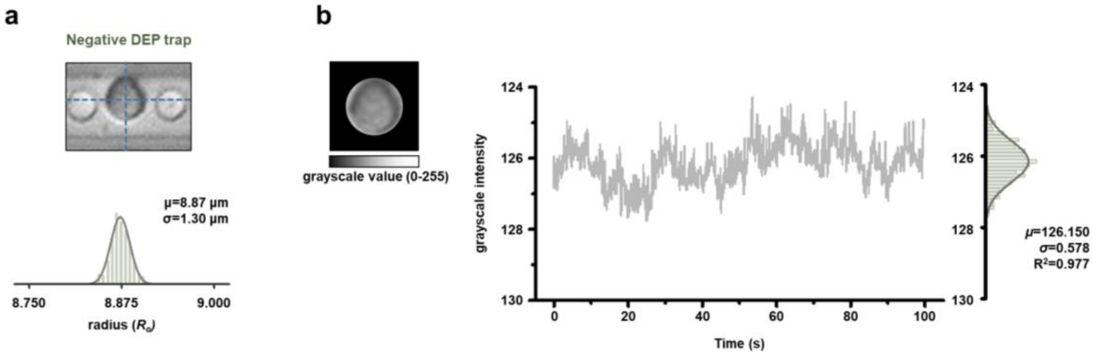

Figure S8. Characterization of cell trapping motion (AC 2 $V_{P\text{-}P}$, 1 kHz) using the modified grayscale measurement method: (**a**) Statistical radius of the artificial window with R_o=8.87 ± 1.30 μm. (**b**) The grayscale value fluctuation with thermal energy.

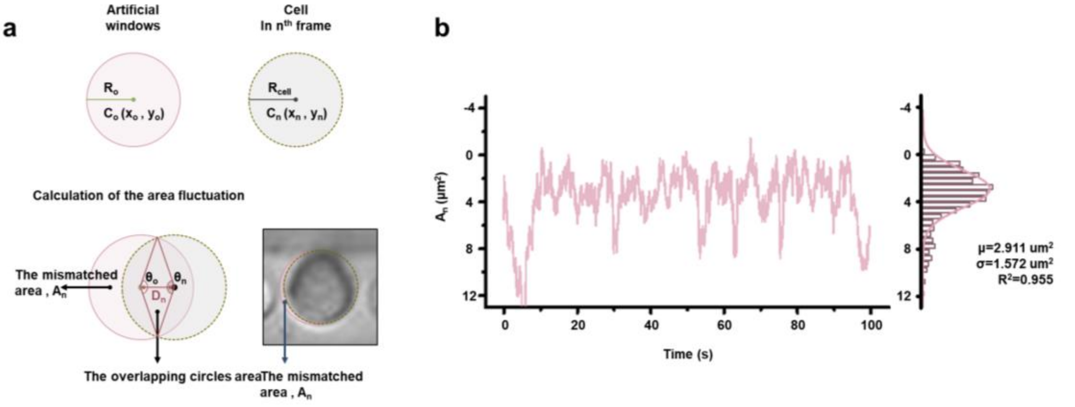

Figure S9 (**a**) Schematic illustration representing the mismatched area (A_n). (**b**) Fluctuating mismatched regions from the time-sequential images that record cellular movement.

Supplementary References

1. Henslee, E.A.; Sano, M.B.; Rojas, A.D.; Schmelz, E.M.; Davalos, R.V., Selective concentration of human cancer cells using contactless dielectrophoresis. *ELECTROPHORESIS* **2011**, *32*, 2523–2529.
2. Davies, E.R. *Machine Vision: Theory, Algorithms, Practicalities*. Elsevier Science: San Francisco, CA, USA, 2004.
3. Atherton, T.J.; Kerbyson, D.J. Size invariant circle detection. *Image Vision Comput.* **1999**, *17*, 795–803.
4. Yuen, H.K.; Princen, J.; Illingworth, J.; Kittler, J. Comparative study of Hough Transform methods for circle finding. *Image Vision Comput.* **1990**, *8*, 71–77.
5. Leon-Garica, A. *Probablity and Random Process for Electrical Engineering*, 2nd ed.; Addison-Wesley Publishing Company: New York, NY, USA, 1994.

© 2018 by the authors. Submitted for possible open access publication under the terms and conditions of the Creative Commons Attribution (CC BY) license (http://creativecommons.org/licenses/by/4.0/).
